# Randomized controlled trial to evaluate a prevention program for frail community-dwelling older adults: a D-SCOPE protocol

**DOI:** 10.1186/s12877-018-0875-3

**Published:** 2018-08-27

**Authors:** Deborah Lambotte, Liesbeth De Donder, Ellen E. De Roeck, Lieve J. Hoeyberghs, Anne van der Vorst, Daan Duppen, Michaël Van der Elst, Bram Fret, Sarah Dury, An-Sofie Smetcoren, Martinus J. M. Kardol, Sebastiaan Engelborghs, Peter Paul De Deyn, Nico De Witte, Jos M. G. A. Schols, Gertrudis I. J. M. Kempen, G. A. Rixt Zijlstra, Jan De Lepeleire, Birgitte Schoenmakers, Dominique Verté, Eva Dierckx

**Affiliations:** 1 Department of Educational Sciences, Vrije Universiteit Brussel, 2, Pleinlaan, Brussels, 1050 Belgium; 2 Laboratory of Neurochemistry and Behavior, University of Antwerp, 1, Universiteitsplein, Wilrijk, 2610 Belgium; 3 Department of Clinical and Lifespan Psychology, Vrije Universiteit Brussel, 2, Pleinlaan, Brussels, 1050 Belgium; 4 Faculty of Education, Health and Social Work, University College Ghent, 80, Keramiekstraat, Ghent, 9000 Belgium; 5 Department of Health Services Research, Care and Public Health Research Institute (CAPHRI), Maastricht University, P.O. Box 616, Maastricht, 6200, MD The Netherlands; 6 Department of Public Health and Primary Care, University of Leuven, 33, Kapucijnenvoer, Leuven, 3000 Belgium; 7 Research Foundation Flanders (FWO), 5, Egmontstraat, Brussels, 100 Belgium; 8 Department of Family Medicine, Care and Public Health Research Institute (CAPHRI), Maastricht University, P.O. Box 616, Maastricht, 6200, MD The Netherlands

**Keywords:** Randomized controlled trial, Frailty, Community-dwelling, Prevention, Detection, Care and support

## Abstract

**Background:**

Frail community-dwelling older adults, whom might experience problems regarding physical, cognitive, psychological, social and environmental factors, are at risk for adverse outcomes such as disability, institutionalization and mortality. People in need of help do not always find their way to care and support services and are left undetected. The aim of the D-SCOPE project is to detect frail community-dwelling older adults who previously went unnoticed and to improve their access to care and support. Goal is to increase their frailty-balance, quality of life, meaning in life, life satisfaction, mastery, community inclusion and ageing well in place.

**Methods/design:**

The study is a prospective, longitudinal randomized four-armed controlled trial with follow-up at 6 months. The study group aims to include 900 community-dwelling older adults aged 60 years and over from 3 municipalities in Flanders (Belgium). While selecting the study group, risk profiles for frailty will be taken into account. Participants will be randomly selected from the census records in each municipality. Data will be collected prospectively at baseline (T0) and at follow-up, 6 months after baseline (T1). At baseline, participants who are at least mild frail on one of the 5 domains of frailty (CFAI-plus) or feel frail based on the subjective assessment of frailty will be randomly assigned to (1) the study group or (2) the control group. A mixed method design with the inclusion of quantitative and qualitative data analyses will be used to evaluate the efficacy and experiences of the detection and prevention program on frailty.

**Discussion:**

The study will contribute to an innovative vision concerning the organization of care and support, and a timely and accurate detection and support of community-dwelling older adults at risk for frailty.

**Trial registration:**

This trial was registered at ClinicalTrials.gov, on May 26, 2017, identifier: NCT03168204.

## Background

Frailty is a common phenomenon in community-dwelling older adults. Research indicates that the average prevalence for multidimensional frailty is 13.6% and 33.5% for prefrailty in community-dwelling older adults [1]. Frailty increases with age [1, 2]. For example, a systematic review on the prevalence of frailty indicates that the prevalence for oldest-old people is 15.7% (80–84 years) and 26.1% (≥ 85 years) [1]. Within an aging society, more and more persons are confronted with frailty and the demand for care and support increases [3–6]. Although frailty has mainly been approached as a physical issue [7, 8], different researchers point to the necessity to operationalize frailty as a multidimensional and dynamic concept that considers the complex interplay of physical, cognitive, psychological, social and environmental factors [9–12]. Not only researchers identify frailty in a multidimensional way, older adults themselves experience frailty as more than merely a physical issue as well [13].

As older adults become frail, different dimensions of their lives such as their quality of life and feelings of control (e.g. mastery) may be affected [14–16], and their risk for adverse outcomes such as hospitalization and institutionalization increases [17–19]. A study by Rockwood et al. [19] indicates that frailty is one of the most important predictors of death and institutionalization. Governments are implementing a proactive care approach in order to prevent or delay (the high costs of) such institutionalization and other adverse outcomes, and stimulate older adults to stay in their own environment as long as possible with good quality of life [20]. This so-called policy on aging in place is in line with the wish of the majority of older adults [21], even when they need care and support, have economic difficulties or live in inadequate housing or deprived environments [22].

People in need of care and support do not always find the appropriate services and are often left undetected [23]. Nowadays in several European countries, also in Belgium, there is insufficient continuity and coherence between the different care and support services in the community [24]. Research indicates that 6.4% of Flemish older adults in need of care and support do not receive any care at all [25]. As a result, the problems and needs of older adults are frequently not recognized or treated in time, leading to a decline of their autonomy and quality of life. Furthermore, current initiatives to proactively identify health and social problems in (frail) older adults insufficiently address needs of (frail) older people [26]. This suggests a need for rethinking the organization of the support and care system [27]. For instance, empowering older adults to manage their own health and social issues and improving their access to community care and support needs to be ameliorated [28]. Therefore, early detection of frailty and tailored care and support are of main importance [29].

Within the detection and support of frail older adults, a critical consideration should be made. Frailty in older adults does not necessarily have negative consequences in daily life, especially when the right care and support is present. This suggest that besides measuring the deficits of frailty, there is also a need to take into account the strengths and resources of older adults [30]. Therefore, we prefer to use balancing factors and the frailty balance as terminology. The latter is in line with Baltes and Smith [31] who suggest the recognition of two faces of human aging, including both the gains and the losses. Such gains might also be seen in the context of losses, as older adults may unfold unexpected substitute skills, collaborative relationships or creative strategies to overcome limitations [32]. For instance, two individuals with a similar frailty level or profile can have different needs of support because their ‘frailty-balance’ is different [33]. Thereby, they might differ in terms of autonomy, resilience, social contacts and received informal and formal care. Interventions are necessary to close of diminish the distance of the gap between gains and losses, and to restore the frailty-balance [33]. Furthermore, older adults need to be supported in using and further develop their own competences [30].

Findings from aforementioned studies, additional literature reviews [34–36] and preliminary studies [29, 37–42] led to the design of a multidimensional detection and prevention program for frail community-dwelling older adults (D-SCOPE) aimed to improve access to care and support. Research indicates that for both men and women, increased age, having no partner, having moved in the previous 10 years, having a lower educational level and having a lower household income are risk characteristics for frailty [29]. Furthermore, different risk profiles for frailty in older adults exist according to gender and the type of frailty (physical, psychological, social, environmental and total frailty). In addition to frailty, it is also important to identify and strengthen the competences and resources of older adults [30, 40]. For example, a literature review concerning the social environment of older adults indicates that different aspects of the social environment such as subjective neighborhood characteristics are protective for frailty in community-dwelling older adults [34]. Another systematic review found a high level of physical activity and being married to be protective against developing limitations in ADLs in community-dwelling persons aged 75 years and over [35].

This article describes the design of the Randomized Controlled Trial (RCT) aimed to evaluate a detection and prevention program on frailty (D-SCOPE), which will create a continuum of care and support for frail community-dwelling older adults, from early detection, over intervention, to follow-up. The D-SCOPE frailty program intends to develop methods to easily, accurately and timely detect and prevent a negative frailty-balance in older adults. The intervention will include tailored care and support and long term care follow-up. The RCT will explore if the D-SCOPE frailty program improves the quality and efficacy of care and support given to frail community-dwelling older adults, which ultimately would increase their quality of life, meaning in life, life satisfaction, mastery, community inclusion and ageing well in place.

The objectives of this trial are to conduct:

1. An effect evaluation to determine if the D-SCOPE frailty program
  - detects frail community-dwelling older adults who otherwise would have remained undetected (i.e., older adults who are at least mild frail on one of the 5 domains of frailty of the CFAI-plus or feel frail based on the subjective assessment of frailty, who do not receive the necessary care and support, by using risk profiles based on age, gender, marital status, migration background and being moved in the past 10 years [29])
  - guides frail community-dwelling older adults towards appropriate care and support (by recognizing, valorizing and strengthening their competences, strengths and resources)
  - prevents that care and support is discontinued (by the older person itself, the care and support organization, discontinuity or care selection by the organization) and thus reduce dropout
  - improves the frailty-balance of community-dwelling older adults (i.e., effect on frailty, balancing factors and outcomes)
2. A process evaluation to determine the obstructing and facilitating components when implementing the D-SCOPE frailty program:
  - On the micro-level: concerning the individual capacities of key-actors (volunteers, municipal health and social care professionals, etc.) such as motivation, needed outcomes, required training and features of older adults (financial vulnerability, care expenditures, etc.)
  - On the meso-exo-level: concerning interpersonal relations, management, administrative support, professional networks, etc.
  - On the macro-level: concerning the broader care system, present care and support organizations in the network, political recommendations, available resources, etc.

## Methods/design

### Study design

**Fig. 1:**
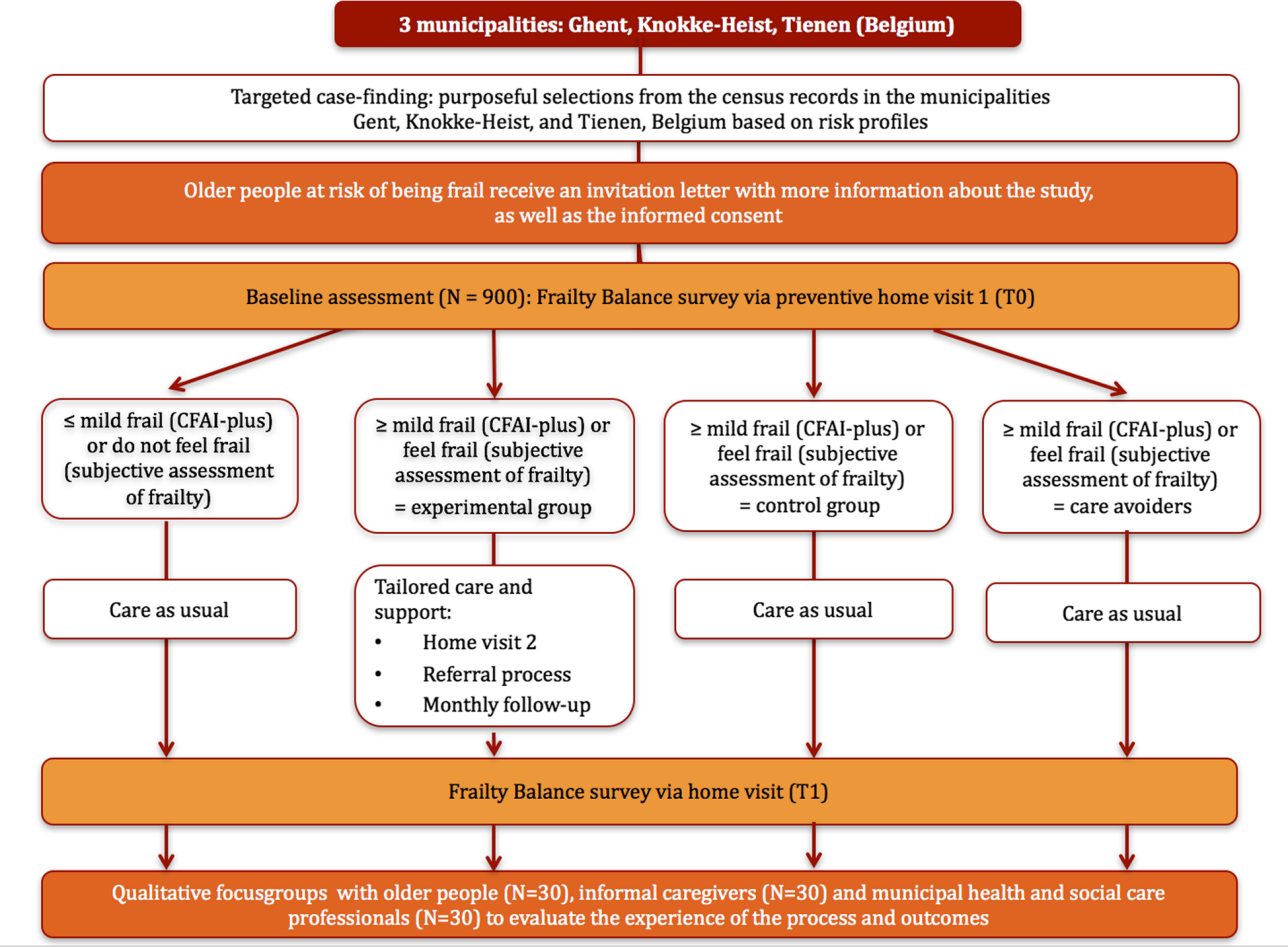
Flow diagram of the D-SCOPE frailty program

The D-SCOPE frailty program concerns a Randomized Controlled Trial (RCT). The RCT will compare usual care with an intervention that include tailored care and support and long-term care follow-up. Figure 1 presents an overview of the study design. The D-SCOPE frailty program will start with targeted case-finding, which refers to the selections from the census records based on eligibility criteria [29]. Older adults will receive an invitation letter explaining the purpose of the study, the way on which the study will be conducted and the expectations towards people who agree to participate. A trained volunteer or a researcher will contact them in person and will inform them face to face about the study. Participants will also receive the informed consent form and a letter for the general practitioner explaining the D-SCOPE frailty program. Respondents will have the opportunity to ask questions if anything would remain unclear. Older adults willing to participate will undergo the baseline assessment (T0) after signing the informed consent.

Older adults who are at least mild frail on one of the 5 domains of frailty (CFAI-plus) or feel frail based on the subjective assessment of frailty will be randomly assigned to either (1) the experimental group or (2) the control group. Older adults with no-to-low frailty (CFAI-plus) or who do not feel frail (subjective assessment of frailty) will be grouped into a third group. A fourth group will include frail older adults willing to participate in the T0 and T1 assessment but not in the intervention part. All groups except for the experimental group will receive care as usual.

All older participants will be assessed after a 6 months’ period. The study will include an effect evaluation and a process evaluation of the RCT. The effect study will be conducted using a quantitative evaluation of the outcome measures for frail community-dwelling older adults. The process evaluation will be performed by a quantitative monitoring of the experimental group (including follow-up telephone interviews) and qualitative focus groups with older adults, informal caregivers and municipal health and social care professionals in each municipality.

The baseline assessment T0 will begin in June 2017 until October 2017. The study assessment T1 will begin in December 2017 until April 2018. The intervention will take place between June 2017 and March 2018, between the baseline assessment T0 and the study assessment T1. The qualitative evaluation will take place in March and April 2018. The data analysis will take place between April 2018 and June 2018.

The content of the program has been developed in close collaboration with representatives of different home care and support levels, i.e. general practitioners, home care organizations, social service of the municipalities, home nurses, older people’s organizations, centers of expertise in housing and care, care insurances companies, universities, etc.

### Setting

The RCT will be conducted in three municipalities in Flanders (Belgium): Knokke-Heist, Ghent and Tienen (*N* = 900, 300 in each municipality). Each municipality was chosen due to their specific characteristics (Table 1). First, the municipalities differ in terms of socio-economic environment [43]. Knokke-Heist is defined as a coastal town, Ghent as a big city and Tienen as a medium-sized town. Second, demographically and regarding welfare and health, Knokke-Heist has been confronted with a sharp increase of older adults despite a low number of places in residential care and a low number of informal care and home care. Like Knokke-Heist, Tienen has more older adults (both 65+ and 80+) than the percentage of the Flemish region. Finally, the population in Ghent has a lower average income in comparison with the Flemish average.

**Table 1:** Characteristics of the 3 localities [74–76]

	Knokke-Heist [74]	Ghent [75]	Tienen [76]	Flemish region [74–76]
Demographic data (2016)				
Total number of inhabitants	33,311	257,029	34,185	6,477,804
Population growth since 2005	98.5	111.3	107.7	107.2
Total number of older inhabitants	11,310	42,706	7,243	1,265,666
Older population growth since 2005	128.5	102.1	111.2	117.9
% older adults (65 years and over)	34.0%	16.6%	21.2%	19.5%
% older adults (80 years and over)	9.9%	5.5%	6.5%	5.9%
Socio-economic data (2013)				
Average income per capita in €	23,374	17,477	18,479	18,163
Welfare and health data				
Number of places in residential care in 2016 per 1000 older adults (>65y)	58	84.6	90.2	76
Number of entitled informal and home care in 2015 per 1000 older adults (>65y)	56.3	69	132.1	98.2

### Ethics

This study protocol was reviewed and approved by the medical ethics committee of the Vrije Universiteit Brussel, Brussels, Belgium (reference number: B.U.N. 143,201,630,458). Prior to the baseline assessment and start of the intervention in the experimental group, written consent will be obtained from all participants.

### Participants and eligibility criteria

Study participants will be community-dwelling older adults aged 60 years and over. In order to explore the most efficient selection strategy to detect frail older adults, two randomized selections, with replacement addresses, from the census records will take place in each municipality. Each randomized selection in each municipality will include 150 participants. The two stratified samples will be based on previous research on risk profiles for frailty [29]**.** Risk characteristics for frailty are gender, age, marital status, moved in the past 10 years and migration background. In the first sample (*n* = 450) older participants will need to fulfill at least one criterion. This implies that the participants will be women or aged 70 years and over or not have a partner or have moved in the past 10 years or will have a migration background. In the second sample (*n* = 450) all older participants will need to fulfill all selection criteria. This implies that older participants will be aged 70 years and over, have no partner, and moved last 10 years. The second sample will exclude the variable migration background due to too small samples within the three selected municipalities.

Exclusion criteria will be current hospitalization, institutionalization, when the older participant himself or his/her informal caregiver indicates that the older participant is not able to participate or if the interviewer notes that the older participant is cognitively not capable to provide adequate answers.

Older adults will be included in the RCT if they are at least mild frail on one of the 5 domains of the CFAI-plus (i.e., ≥ 25 for physical frailty, ≥ 12.52 for cognitive frailty, ≥ 20 for psychological frailty, ≥ 37.5 for social frailty and ≥ 5 for environmental frailty) or feel frail based on the subjective assessment of frailty (i.e. at least agree with the statement), and accept to participate in the intervention.

### Randomization

Eligible participants will be randomly assigned directly after the baseline assessment by the principal researcher, using computer-generated randomization to either the control or the experimental group.

### Intervention

The intervention contains several steps in order to empower older adults and improving their access to care and support [28]. Older participants assigned to the experimental group after the first home visit containing the T0 baseline assessment will be contacted by a professional from the social service of the municipality for a second home visit. These professionals will already be experienced with conducting home visits and will receive training and instructions concerning multidimensional frailty, frailty-balance and taking into account the strengths and competences of older adults and their informal caregivers. During the second home visit, the professional from the social service of the municipality will further explore the older adult’s competences, needs and preferences. Based on the results of the baseline assessment and on the results of the second home visit, the professional from the social service of the municipality will propose a type of intervention. The decision and organization of tailored care and support will be made together with the older participant and his/her environment. The older participant will be accompanied in the referral once decided in which organization/form of intervention the older participant will participate in order to reduce dropout. The older participant will receive tailored care and support whereby his competences, strengths and resources will be supported [30]. The intervention will depend on the availability of the care and support services in the municipality, and could be formal (e.g., home care) or informal (e.g., activities of an older adult’s association). A professional from the social service of the municipality will monitor which care the participant receives, when the older person cancels the care and support and if everything is going according to his/her wishes. This will be done monthly by telephone.

### Measurements

Table 2 presents the outcomes of the effect and process evaluation.

**Table 2:** SPIRIT diagram outlining schedule of enrolment, intervention and assessments for study participants

Time points		Enrolment	Baseline assessment T0	Intervention	Quantitative monitoring	Monthly follow-up	Study assessment T1	Qualitative evaluation
Introduction/preparation	Screening eligibility criteria	x						
	Information letter	x						
	Informed consent	x						
Study groups	Experimental group		x	x	x	x	x	x
	Control group		x				x	x
	Care avoiders		x				x	x
	Group ≤ mild frail (CFAI-plus) or do not feel frail (subjective assessment of frailty)		x				x	x
	Informal caregivers							x
	Municipal health and social care professionals							x
Primary outcome measures	Quality of life		x				x	
	Meaning in life		x				x	
	Life satisfaction		x				x	
	Mastery		x				x	
	Community inclusion		x				x	
	Ageing well in place		x				x	
Secondary outcome measures	Multidimensional frailty		x				x	
	Physical phenotype of frailty		x				x	
	Feeling frail		x				x	
	Resilience		x				x	
	Coping		x				x	
	Help needed for activities in daily life		x				x	
	Informal and formal care		x				x	
	Medical care		x				x	
	Leisure time		x				x	
	Neighborhood		x				x	
	Future perspective		x				x	
	Life events		x				x	
Additional variables	Socio-demographic variables		x				x	
	Socio-economic situation		x				x	
Process measures	Amount of intended target group that participated in the second home visit/started the intervention				x			
	Amount and types of delivered intervention(s)				x			
	Number refuses, dropouts and completions				x			
	Logbook				x			
	Reasons for refusal/dropout					x		
	Satisfaction of intervention					x		
	Satisfaction D-SCOPE frailty program							x
	Experiences care/support processes							x
	Components that support or inhibit the implementing process of the D-SCOPE frailty program							x

### Effect evaluation

#### Primary outcome measures

The primary outcomes of the effect evaluation will be quality of life, meaning in life, life satisfaction, mastery, community inclusion and ageing well in place.

*Quality of life* will be measured by the use of one item from the abbreviated version of the World Health Organization Quality of Life (WHOQOL-BREF) [44]. *Meaning in life* will be evaluated with the Meaning in Life Questionnaire (MLQ) and will assess perceived meaning in life by the use of 5 items [45]. *Life satisfaction* will be measured by using the Satisfaction with Life Scale, a validated scale which measures global life satisfaction [46]. To assess *mastery*, a questionnaire which evaluates to what extent people feel they exert control over existing circumstances of their lives with 4 items will be used [47]. In addition, one self-constructed item will assess mastery in relation to others [48]. *Community inclusion* will be measured by using 1 item from the Community Integration Measure (CIM) and will ask the participants to what extent they feel like part of the community [49]. *Ageing well in place* will be assessed using a self-constructed question and will explore to what extent the older participant feels he/she lives at home in a qualitative way. Older participants will also be asked to rate the outcomes quality of life, meaning in life, autonomy and community inclusion on a scale from 0 to 10.

#### Secondary outcome measures

Secondary outcomes will be multidimensional frailty, physical phenotype of frailty, feeling frail, balancing factors (i.e., resilience, coping, help needed for activities in daily life, informal and formal care, medical care, leisure time, neighborhood), future perspective and life-events.

The Comprehensive Frailty Assessment Instrument (CFAI-plus) will measure *multidimensional frailty* [12, 50]. The physical domain evaluates the general physical health (e.g., walking up a hill or stairs); the psychological domain assesses mood-disorders and emotional loneliness (e.g., feeling pressure); the social domain contains social loneliness and social support (e.g., there are enough people I feel close to); and the environmental domain evaluates conditions of inadequate housing using (e.g., my house is in a bad condition). Cognitive frailty was recently added to the CFAI and evaluates cognitive functioning (e.g., memory problems) [50]. The Fried’s phenotype of frailty (slow mobility, weakness, weight loss, decreased activities and exhaustion) [7] will be used to assess the physical phenotype of frailty as well as the questionnaire of Op het Veld et al. [51]. For weakness and slow mobility, 180 older participants will do the physical tests included in the Fried’s phenotype of frailty. The *subjective feeling of frailty* will be assessed with a self-constructed question which explores to what extent the participant agrees with the statement ‘I feel frail’. Older participants will also be asked to rate their subjective feeling of being frail on a scale from 0 to 10. *Resilience* will be measured by using the Connor-Davidson Resilience Scale (CD-RISC2), which is an abbreviated 2-item version of the original Scale [52]. *Coping* will be measured by using 6 items (i.e., active coping, positive reframing, acceptance, religion, emotional support and self-distraction) of the BRIEF Cope Carver scale [53]. By proposing statements, older participants need to answer to what extent they would react like this in a stressful or difficult situation. *Help needed for activities in daily life* will be measured by asking if older participants need help with 8 activities of daily life (i.e., personal care, household tasks, personal displacements, administrative and financial management, social company and support, grocery shopping, chores and supervision), and to what extent the help they receive for these activities is sufficient. These questions are adapted from the questionnaire of the Belgian Ageing Studies (BAS) [54]. *Informal and formal care* will be assessed by asking older participants if they receive care from 7 informal (e.g., children, neighbors, friends) and 13 formal caregivers (e.g., home nursing), and if they are satisfied with the help they receive from these caregivers. These questions are adapted versions of the BAS-questionnaire [54]. *Medical care* will be measured by asking how many times the participants needed to go to a general practitioner, a hospital, residential setting and/or rehabilitation center over the past 6 months (day care/overnight stay). These questions are adapted from the Health Interview Survey [55]. In addition, the participants will be asked when they visited a general practitioner for the last time. Also different aspects of the environment will be assessed. First, the *social environment* will be administered by using 3 items from the social cohesion dimension of the Neighborhood Scale [56]. Second, the *physical environment* will be explored by using 4 items from the BAS-questionnaire [54] as well as from the Neighborhood Environment Walkability Scale [57]. In terms of *participation*, leisure time will be measured by using an adapted question with 8 items derived from the BAS questionnaire [54]. This question will examine how often the participants perform following activities: giving care or support, voluntary work, activities at home, sport activities outside a club, cultural activities, activities in an organization, going to a bar/restaurant/shopping center/trips, attend training. Also, low-key social participation will be examined by using 2 items from the questionnaire of Oswald and Konopik [58]. *Future perspective* will be assessed by using a self-constructed question and will explore to what extent the participant has things to look forward to. Finally, the occurrence of *life-events* will be assessed by using a shortened version (11 items) of the Geriatric Adverse Life Events Scale (GALES) [59, 60].

#### Additional measures

Several variables will be assessed in order to provide insight information concerning the study population, and to interpret outcomes of the study. These are the socio-demographic variables, assessed during the process of screening for eligibility: age, gender, country of birth, educational level, marital status and moved last 10 years [29, 54]. Additionally, also nationality will be assessed as well as the socio-economic situation (net monthly household income) [54].

The questionnaires will be available in Dutch and in French. Questions from existing instruments will be translated (if not already validated in the respective language) using a team translation approach called the Translation, Review, Adjudication, Pre-Testing and Documentation (TRAPD) translation model [61]. Team approaches provide the richest output in options to choose from for translation. Another advantage of this method is the acquisition of balanced critique and a more fundamental choice between different versions [62]. In the TRAPD model, several translators will make independent parallel translations of the same questionnaire [61]. Thereafter, the translators and translation reviewers will go through the entire questionnaire discussing versions and agreeing on a final review version. The version produced trough discussion will move on to adjudication. The survey will also be screened by “Wablieft”, an organization who will check the accessibility and clarity of the survey taking into account the target group, (possibly frail) older adults.

#### Process evaluation

In order to evaluate the quality and sustainability of the D-SCOPE frailty program a quantitative questionnaire will be used during the intervention to measure the number of older adults that participated in the second home visit (1), started the intervention (2) and, dropped-out during the intervention (3). A professional from the social service of each municipality will also keep track of a logbook. In this logbook the amount of contacts in the intervention, the offered informal and formal care and support, satisfaction about the offered care and support and the problems encountered during the intervention will be registered. The reasons for refusal/dropout before the start as well as during the intervention will be evaluated as well.

In addition, after T1, in each municipality 3 focus groups will be organized: one with older adults who participated in one of the four groups, one with informal caregivers and one with professionals participating in the D-SCOPE frailty program. Because in complex interventions social or behavioral processes are difficult to explore using quantitative methods alone [63] the use of an additional qualitative research design will be helpful in providing valuable new insights. The goal is to determine the participant’s opinions concerning the added value of the program and to identify components that support or inhibit the process of implementing the D-SCOPE frailty program. The focus groups will be held by a semi-structured interview schedule, developed following a literature review and input from the D-SCOPE consortium, consisting of researchers from different research areas from different universities:

- Satisfaction with the D-SCOPE frailty program
- Experiences of the care/support processes
- Identification of components that support or inhibit the process of implementing the D-SCOPE frailty program: the extent to which success factors or problems were encountered while applying the program.

#### Data gathering

Data on baseline characteristics, frailty, balancing factors and outcomes will be collected to evaluate the effect of the intervention with questionnaires at two points in time: T0 and T1 (6 months after inclusion). Participants in the study will concern all groups; the experimental group of the RCT, the control group of the RCT, older participants with no-to-low frailty (CFAI-plus) or who do not feel frail (subjective assessment of frailty) and care avoiders. Trained volunteers or researchers will collect the T0 data. A professional from the social service of the municipality will receive all completed questionnaires and informed consents from the baseline assessment after the first home visit and will register the completed questionnaires in a specific designed computer program named Qualtrics. The research coordinator, who is responsible for the randomization to the control and experimental group, will be the person who can consult the results and will communicate to the municipality which respondents are randomized in the experimental group. The municipality will arrange that the experimental group receives a second home visit by a professional from the social service of the municipality, search an appropriate intervention and do the follow-up. After 6 months, trained volunteers and researchers will collect the T1 data. A professional from the social service of the municipality will again receive all completed questionnaires from the T1 assessment and will register them in the specific designed computer file. At the end, the research coordinator will consult the data.

#### Power analysis

As we have no clear view yet on all aspects, factors, scales, outcomes, statistical analyses, etc., it is not feasible to run detailed power analyses a priori. However, according to Cohen [64] and when using the online a priori sample size calculator for independent sample t-tests [65]; the minimum sample size per group (experimental as well as control group) with a probability level of .05, an anticipated Cohen’s d effect size of 0.5 (medium) and a desired statistical power of 0.8 will be 64. To find differences with a small effect size (cohen’s d = 0.2) between the 2 groups a total n of 788 (394 in each group) is required.

As is our longitudinal design is concerned, we have based our a priori estimation on a study of Fabricotti et al. [66]. They expect a 10% loss to follow up (due to mortality, re-housing, impossibility or unwillingness to participate further) between T0 and T1. By including 220 older adults in both the experimental and control group, they state that their sample is sufficient to detect changes. Assuming an average effect size of 0.5 and significance of 5%, this gives a power of 0.997. They further argue that if a small effect size is expected of 0.3 with a significance of 5%, this still supplies sufficient power at 0.837. Interfering variables will also play a role. At an average effect size (f2) of 0.15 and significance of 5%, assuming five independent variables, the power is 0.97. Even with 15 independent variables, the power remains sufficient at 0.856.

So, in sum, it was decided to aim for 900 community-dwelling older adults aged 60 years, equally divided over the municipalities Knokke-Heist, Ghent and Tienen in Flanders, Belgium; so 300 community-dwelling older adults will participate in each municipality.

#### Blinding

Older participants, interviewers performing the baseline assessment and researchers doing the outcome assessment and data analysis will be blinded to group allocation. It won’t be possible to blind the research coordinator and municipal health and social care professionals performing the intervention to group allocation. Interviewers won’t be blinded when doing the follow-up.

### Analysis of the data

#### Effect evaluation

The experimental and control groups will be described at both time points with descriptive statistics. Similarity of characteristics between the two groups will be assessed by means of independent sample t-tests or chi-square tests. Differences in measurements between T0 and T1 will be assessed by means of repeated measures ANOVA’s. In order to explore which combination of resources lead to a higher quality of life, meaning in life, life satisfaction, mastery, community inclusion and ageing well in place in frail community-dwelling older adults, different interaction models with balancing factors as moderators will be tested. We will explore which balancing factors are moderators for having a good quality of life, meaning in life, life satisfaction, mastery, community inclusion and ageing well in place, despite being frail. We will explore the hypothesis that frail older adults who have the adequate resources (positive frailty-balance) have a better chance for aforementioned positive outcomes than frail older adults with a negative frailty-balance. For each measure, regression analyses will be performed with the T1 scores as the dependent variable, the research group (experimental vs. control) as independent variable of interest, and demographic variables and differences in baseline characteristics as co-variates. Multivariate analyses will be performed in order to answer the research questions.

#### Process evaluation

All qualitative interviews will be audio-recorded and transcribed verbatim. Interviews will be analyzed by the use of thematic content analysis. In order to increase the credibility of the findings, the coding frames and strategies will be subject to systematic review by the two principal investigators and refined through a process of consensus. Findings from each focus group will be analyzed separately to have a cross-sectional perspective, and only after separate analysis has taken place the focus groups will be combined for a comparative analysis. This generates cross-sectional descriptions of each municipality and enables a comparative view capturing similarities and differences between localities.

All quantitative data will be analyzed with SPSS [67] and all qualitative data will be analyzed using the MAXQDA software package [68].

## Discussion

In order to detect and prevent frailty worsening in community-dwelling older adults, a program on detection and prevention is needed, involving targeted case-finding, individualized assessment, tailor-made interventions and repeated short term follow-up. In the upcoming years, the aging population will increase the challenges on health care systems and consequently the management of community care and support needs to be reconsidered. By introducing the D-SCOPE frailty program, we aim to provide an efficient structure for a timely and accurate detection of frailty-imbalance in community-dwelling older adults and for the organization and delivery of efficient and effective care and support.

Some challenges will be taken into account. Research indicates that the implementation of integrated care programs is challenging and difficult [69]. For example, frail individuals who receive care and support often lose control over their own lives and receive little opportunity to shape their own care. Furthermore, when integrated care interventions are successfully implemented in one specific setting, the dissemination on a wider scale remains challenged [70]. Several activities were and will continue to be organized to face these challenges. The D-SCOPE frailty program has been designed in close collaboration with different actors specialized in care and support for older adults, which along with the process evaluation and future protocol meetings are intended to ensure the quality and sustainability of the program.

The D-SCOPE program has a number of strengths, which makes the study relevant for science and practice. First, this program will not focus of the total older population of the municipality but will target older adults with an increased risk for frailty. This targeted case-finding will permit the organization of well-coordinated, targeted and comprehensive home and community care, which is a key factor to maintain frail older adults at home [71]. Second, this program will create a tailored delivery system of care and support by the use of a frailty-balance approach. Two individuals with the same frailty may have a total different quality of life, autonomy, etc. due to their strengths, resources and skills accumulated or lost over time [72]. The D-SCOPE program acknowledges the fact that need of care, support and empowerment is highly personal [33]. Moreover, current frailty instruments often lead to false positives [73]. With the development of a frailty-balance instrument “diagnostic” accuracy can improve as only those older adults who are in need of care and/or support will be included in an integrated care and support trajectory.

In summary, the D-SCOPE program will contribute to the creation of a continuum of care and support for frail older adults who often remain undetected. It will enhance the organization and transfer of care and support, which will have advantages for the individuals as well as for society. Specifically, the study is expected to show positive results on the quality of life, life satisfaction, meaning in life, autonomy and community inclusion of frail community-dwelling older adults as a consequence of tailor-made interventions.

## Abbreviations

BAS: Belgian Ageing Studies
CD-RISC2: Connor-Davidson Resilience Scale
CFAI-plus: Comprehensive Frailty Assessment Instrument
CIM: Community Integration Measure
D-SCOPE: Detection, Support and Care for older people, Prevention and Empowerment
GALES: Geriatric Adverse Life Events Scale
MAXQDA: Qualitative Data Analysis Software
MLQ: Meaning in Life Questionnaire
RCT: Randomized Controlled Trial
SPSS: Statistical Package for the Social Sciences
TRAPD: Translation, Review, Adjudication, Pre-testing and Documentation translation model
WHOQOL-BREF: World Health Organization Quality of Life abbreviated version

## References

[1] Collard RM, Boter H, Schoevers RA, Oude Voshaar RC. Prevalence of frailty in community-dwelling older persons: a systematic review. J Am Geriatr Soc. 2012; 10.1111/j.1532-5415.2012.04054.x. 10.1111/j.1532-5415.2012.04054.x 22881367

[2] Lee DR, Kawas CH, Gibbs L, Corrada MM. Prevalence of Frailty and Factors Associated with Frailty in Individuals Aged 90 and Older: The 90+ Study. J Am Geriatr Soc. 2016; 10.1111/jgs.14317. 10.1111/jgs.14317 PMC12893761 27590837

[3] Bolin K, Lindgren B, Lundborg P. Informal and formal care among single-living elderly in Europe. Health Econ. 2008; 10.1002/hec.1275. 10.1002/hec.1275 17768700

[4] Yang Z, Norton EC, Stearns SC. Longevity and health care expenditures: the real reasons older people spend more. J Gerontol B Psychol Sci Soc Sci. 2003; 10.1093/geronb/58.1.S2. 10.1093/geronb/58.1.s2 12496303

[5] Colombo F, Llena-Nozal A, Mercier J, Tjadens F. Help wanted? Providing and paying for long-term care: OECD Health Policy Studies. Paris: OECD Publishing; 2011.

[6] Jacobs M, Van Tilburg T, Groenewegen P, Broese van Groenou M. Linkages between informal and formal caregivers in home-care networks of frail older adults. Ageing Soc. 2016; 10.1017/S0144686X15000598.

[7] Fried LP, Tangen CM, Walston J, Newman AB, Hirsch C, Gottdiener J, et al. Frailty in older adults: evidence for a phenotype. J Gerontol A Biol Sci Med Sci. 2001; 10.1093/gerona/56.3.M146. 10.1093/gerona/56.3.m146 11253156

[8] Viana JU, Silva SLA, Torres JL, Dias JMD, Pereira LSM, Dias RC. Influence of sarcopenia and functionality indicators on the frailty profile of community-dwelling elderly subjects: a cross-sectional study. Braz J Phys Ther. 2013; 10.1590/S1413-35552013005000102. 10.1590/S1413-35552012005000102 23970115

[9] Armstrong JJ, Stolee P, Hirdes JP, Poss JW. Examining three frailty conceptualizations in their ability to predict negative outcomes for home-care clients. Age Ageing. 2010; 10.1093/ageing/afq121. 10.1093/ageing/afq121 20858672

[10] Bergman H, Ferrucci L, Guralnik J, Hogan DB, Hummel S, Karunananthan S, et al. Frailty: An Emerging Research and Clinical Paradigm—Issues and Controversies. J Gerontol A Biol Sci Med Sci. 2007; 10.1093/gerona/62.7.731. 10.1093/gerona/62.7.731 PMC2645660 17634320

[11] De Witte N, De Donder L, Dury S, Buffel T, Verté D, Schols J (2013). A theoretical perspective on the conceptualisation and usefulness of frailty and vulnerability measurements in community dwelling older adults. Aporia: the Nursing Journal.

[12] De Witte N, Gobbens R, De Donder L, Dury S, Buffel T, Schols J, et al. The comprehensive frailty assessment instrument: development, validity and reliability. Geriatr Nurs. 2013; 10.1016/j.gerinurse.2013.03.002. 10.1016/j.gerinurse.2013.03.002 23608069

[13] Grenier A. Constructions of frailty in the English language, care practice and the lived experience. Ageing Soc. 2007; 10.1017/S0144686X06005782.

[14] Gobbens RJJ, van Assen MALM. The prediction of quality of life by physical, psychological and social components of frailty in community-dwelling older people. Qual Life Res. 2014; 10.1007/s11136-014-0672-1. 10.1007/s11136-014-0672-1 24671672

[15] Kojima G, Iliffe S, Jivraj S, Walters K. Association between frailty and quality of life among community-dwelling older people: a systematic review and meta-analysis. J Epidemiol Community Health. 2016; 10.1136/jech-2015-206717. 10.1136/jech-2015-206717 26783304

[16] Lee W-J, Chen L-K, Peng L-N, Chiou S-T, Chou P (2016). Personal mastery attenuates the adverse effect of frailty on declines in physical function of older people. A 6-year population-based cohort study. Medicine.

[17] Espinoza S, Walston JD (2005). Frailty in older adults: insights and interventions. Cleve Clin J Med.

[18] Ament BHL, de Vugt ME, Verhey FRJ, Kempen GIJM. Are physically frail older persons more at risk of adverse outcomes if they also suffer from cognitive, social, and psychological frailty? Eur J Ageing. 2014; 10.1007/s10433-014-0308-x. 10.1007/s10433-014-0308-x PMC5549203 28804327

[19] Rockwood K, Howlett SE, MacKnight C, Beattie BL, Bergmann H, Hébert R, et al. Prevalence, attributes, and outcomes of fitness and frailty in community-dwelling older adults: report from the Canadian study of health and aging. J Gerontol A Biol Sci Med Sc. 2004; 10.1093/gerona/59.12.1310. 10.1093/gerona/59.12.1310 15699531

[20] Scharlach A. Creating aging-friendly communities in the United States. Ageing Int. 2012; 10.1007/s12126-011-9140-1.

[21] Löfqvist C, Granbom M, Himmelsbach I, Iwarsson S, Oswald F, Haak M. Voices on relocation and aging in place in very old age. A Complex and Ambivalent Matter Gerontologist. 2013; 10.1093/geront/gnt034. 10.1093/geront/gnt034 23626372

[22] Smetcoren A-S, De Donder L, Dury S, De Witte N, Kardol T, Verté D. Refining the push and pull framework: identifying inequalities in residential relocation among older adults. Ageing Soc. 2017; 10.1017/S0144686X15001026.

[23] Willemé P. The Long-Term Care System for the Elderly in Belgium. ENEPRI Research Report n°70. 2010. http://www.ancien-longtermcare.eu/sites/default/files/ENEPRI%20RR%2070%20ANCIEN%20Belgian.pdf. Accessed 5 Jan 2017.

[24] Paulus D, Van den Heede K, Mertens R (2012). Position paper: Organisation of care for chronically ill patients in Belgium. Health Services Research (HSR).

[25] De Witte N, Buffel T, De Donder L, Dury S, Verté D (2010). Care shortages in later life: the role of individual and contextual variables in Flanders. Belgium IJ-SSHS.

[26] Lette M, Baan CA, van den Berg M, de Bruin SR. Initiatives on early detection and intervention to proactively identify health and social problems in older people: experiences from the Netherlands. BMC Geriatr. 2015; 10.1186/s12877-015-0131-z. 10.1186/s12877-015-0131-z PMC4628317 26518369

[27] Nyweide DJ, Anthony DL, Chang CH, Goodman D. Seniors’ perceptions of health care not closely associated with physician supply. Health Aff. 2011; 10.1377/hlthaff.2010.0602. 10.1377/hlthaff.2010.0602 PMC3070266 21289342

[28] Tindale J, Denton M, Ploeg J, Lilie J, Hutchison B, Brazil K, et al. Social determinants of older adults’s awareness of community support services in Hamilton. Ontario Health Soc Care Community. 2001; 10.1111/j.1365-2524.2011.01013.x. 10.1111/j.1365-2524.2011.01013.x 21718377

[29] Dury S, De Roeck E, Duppen D, Fret B, Hoeyberghs L, Lambotte D, et al. Identifying frailty risk profiles of home-dwelling older people: focus on sociodemographic and socioeconomic characteristics. Aging Ment Health. 2016; 10.1080/13607863.2016.1193120. 10.1080/13607863.2016.1193120 27267783

[30] Buntinx F, Paquay L, Fontaine O, Ylieff M, De Lepeleire J (2004). Options for a new procedure for determining care needs in Belgium: an initial exploration. Arch of Public Health.

[31] Baltes PB, Smith J. New Frontiers in the Future of Aging: From Successful Aging of the Young Old to the Dilemmas of the Fourth Age Gerontology. 2003; 10.1159/000067946. 10.1159/000067946 12574672

[32] Hansson RO, Robson SM, Limas MJ (2001). Stress and coping among older workers. Work.

[33] de Blok C, Meijboom B, Luijkx K, Schols J. Demand-based provision of housing, welfare and care services to elderly clients: from policy to daily practice through operations management. Health Care Anal. 2009; 10.1007/s10728-008-0095-7. 10.1007/s10728-008-0095-7 18642082

[34] Duppen D, Van der Elst MCJ, Dury D, Lambotte D, De Donder L. D-SCOPE The Social Environment’s Relationship With Frailty: Evidence From Existing Studies. J Appl Gerontol. 2017; 10.1177/0733464816688310. 10.1177/0733464816688310 28380715

[35] van der Vorst A, Zijlstra GAR, De Witte N, Duppen D, Stuck AE, Kempen GIJM, et al. Limitations in activities of daily living in community-dwelling people aged 75 and over: a systematic literature review of risk and protective factors. PLoS One. 2016; 10.1371/journal.pone.0165127. 10.1371/journal.pone.0165127 PMC5070862 27760234

[36] De Roeck EE, Engelborghs S, Dierckx E. Next generation brain health depends on early Alzheimer disease diagnosis: from a timely diagnosis to future population screening. J Am Med Dir Assoc. 2016; 10.1016/j.jamda.2016.02.015. 10.1016/j.jamda.2016.02.015 26972349

[37] De Roeck E, Ponjaert-Kristoffersen I, Bosmans M, De Deyn PP, Engelborghs S, Dierckx E (2016). Are depressive symptoms in mild cognitive impairment predictive of conversion to dementia? Int Psychogeriatr.

[38] Fret B, Lambotte D, Van Regenmortel S, Dury S, De Witte N, Dierckx E, et al. Socio-demographic, socio-economic and health need differences between types of care use in community-dwelling older adults. International Journal of Care and Caring. 2017; 10.1332/239788217X15027193795897.

[39] Dury S, Dierckx E, van der Vorst A, Van der Elst M, Fret B, Duppen D, et al. Detecting frail, older adults and identifying their strengths: results of a mixed-methods study. BMC Public Health. 2018; 10.1186/s12889-018-5088-3. 10.1186/s12889-018-5088-3 PMC5789734 29378540

[40] van der Vorst A, Zijlstra GAR, De Witte N, Vogel RGM, Schols JMGA, Kempen GIJM, et al. Explaining discrepancies in self-reported quality of life in frail older people: a mixed-methods study. BMC Geriatr. 2017; 10.1186/s12877-017-0641-y. 10.1186/s12877-017-0641-y PMC5659025 29073908

[41] Smetcoren AS, Dury S, De Donder L, Dierckx E, De Witte N, Engelborghs S, et al. Detectie en preventie van kwetsbaarheid: Op zoek naar risicoprofielen voor fysieke, psychische, sociale en omgevingskwetsbaarheid. Tijdschr Gerontol Geriatr. 2017; 10.1007/s12439-017-0241-5. 10.1007/s12439-017-0241-5 29181776

[42] Smetcoren AS, Dury S, De Donder L, Dierckx E (2017). D-SCOPE: naar een positieve kijk op preventive bij kwetsbare ouderen. Geron.

[43] Belfius. Lokale financiën. Sociaaleconomische typologie van de gemeenten. 2007. https://www.belfius.be/publicsocial/NL/Media/Typologie_NEW_nl_tcm_31-36262.pdf Accessed 2 Jul 2017.

[44] World Health Organization (WHO). WHOQOL-BREF Introduction, administration scoring and generic version of the assessment. 1996. http://www.who.int/mental_health/media/en/76.pdf?ua=1. Accessed 5 Jan 2017.

[45] Steger MF, Frazier P, Oishi S, Kaler M. The meaning in life questionnaire: assessing the presence of and search for meaning in life. J Couns Psychol. 2006; 10.1037/0022-0167.53.1.80.

[46] Diener E, Emmons RA, Larsen RJ, Griffin S. The satisfaction with life scale. J Pers Assess. 1985; 10.1207/s15327752jpa4901_13. 10.1207/s15327752jpa4901_13 16367493

[47] Pearlin LI, Nguyen KB, Schieman S, Milkie MA. The life-course origins of mastery among older people. J Health Soc Behav. 2007; 10.1177/002214650704800205. 10.1177/002214650704800205 17583272

[48] Verkerk M. The care perspective and autonomy. Med Health Care Philos. 2001; 10.1023/A:1012048907443. 10.1023/a:1012048907443 11760228

[49] McColl MA, Davies D, Carlson P, Johnston J, Minnes P. The community integration measure: development and preliminary validation. Arch Phys Med Rehabil. 2001; 10.1053/apmr.2001.22195. 10.1053/apmr.2001.22195 11295000

[50] De Roeck EE, Dury S, De Witte N, De Donder L, Bjerke M, De Deyn PP (2018). CFAI-plus: adding cognitive frailty as a new domain to the comprehensive frailty assessment instrument. Int J Ger Psych Accepted.

[51] het Veld LPM O, de Vet HCW, van Rossum E, GIJM K, van Kuijk SMJ, AJHM B. Substitution of Fried’s performance-based physical frailty criteria with self-report questions. Arch Gerontol Geriatr. 2018; 10.1016/j.archger.2017.11.009. 10.1016/j.archger.2017.11.009 29202326

[52] Vaishnavi S, Connor K, Davidson JRT. An abbreviated version of the Connor-Davidson resilience scale (CD-RISC), the CD-RISC2: psychometric properties and applications in psychopharmalogical trials. Psychiatry Res. 2007; 10.1016/j.psychres.2007.01.006. 10.1016/j.psychres.2007.01.006 PMC2041449 17459488

[53] Carver CS. You want to measure coping but your protocol's too long: consider the Brief COPE. Int J Behav Med. 1997; 10.1207/s15327558ijbm0401_6. 10.1207/s15327558ijbm0401_6 16250744

[54] De Donder L, De Witte N, Verté D, Dury S, Buffel T, Smetcoren A-S, Brosens D, Verté E. Developing evidence-based age-friendly policies: a participatory research project. Research Methods Cases. 2014; 10.4135/978144627305013508507.

[55] Wetenschappelijk Instituut Volksgezondheid (WIV). Health Interview Survey 2013. In: Questionnaires 2013. https://his.wiv-isp.be/SitePages/Questionnaires.aspx. Accessed 5 Jan 2017.

[56] Mujahid MS, Roux AVD, Morenoff JD, Raghunathan T. Assessing the measurement properties of neighborhood scales: from psychometrics to ecometrics. Am J Epidemiol. 2007; 10.1093/aje/kwm040. 10.1093/aje/kwm040 17329713

[57] Cerin E, Saelens BE, Sallis JF, Frank LD (2006). Neighborhood environment walkability scale: validity and development. Med Sci Sports Exerc.

[58] Oswald F, Konopik N. Impact of out-of-home activities, neighborhood and urban-related identity on well-being in old age. Z Gerontol Geriatr. 2015; 10.1007/s00391-015-0912-1. 10.1007/s00391-015-0912-1 26066027

[59] Devanand DP, Kim MK, Paykina N, Sackeim HA. Adverse life events in elderly patients with major depression or dysthymic disorder and in healthy-control subjects. Am J Geriatr Psychiatry. 2002; 10.1097/00019442-200205000-00005. 11994213

[60] Seematter-Bagnoud L, Karmaniola A, Santos-Eggimann B. Adverse life events among community-dwelling persons aged 65-70 years: gender differences in occurrence and perceived psychological consequences. Soc Psychiat Epidemiol. 2010; 10.1007/s00127-009-0035-3. 10.1007/s00127-009-0035-3 19305935

[61] Harkness JA. Round 4 ESS translation strategies and procedures. 2008. http://www.europeansocialsurvey.org/docs/round4/methods/ESS4_translation_guidelines.pdf. Accessed 22 Jan 2017.

[62] Guillemin F, Bombardier C, Beaton D. Cross-cultural adaptation of health-related quality of life measures: literature review and proposed guidelines. J Clin Epidemiol. 1993; 10.1016/0895-4356(93)90142-N. 10.1016/0895-4356(93)90142-n 8263569

[63] Lewin S, Glenton C, Oxman AD. Use of qualitative methods alongside randomised controlled trails of complex healthcare interventions: methodological study. BMJ. 2009; 10.1136/bmj.b3496. 10.1136/bmj.b3496 PMC2741564 19744976

[64] Cohen J (1988). Statistical power analysis for the behavioral sciences.

[65] A-priori Sample Size Calculator for Student t-Tests. Soper DS. 2016. http://www.danielsoper.com/statcalc. Accessed 11 Feb 2017.

[66] Fabbricotti IN, Janse B, Looman WM, de Kuijper R, van Wijngaarden JDH, Reiffers A. Integrated care for frail elderly compared to usual care: a study protocol of a quasi-experiment on the effects on the frail elderly, their caregivers, health professionals and health care costs. BMC Geriatr. 2013; 10.1186/1471-2318-13-31. 10.1186/1471-2318-13-31 PMC3648376 23586895

[67] Field A (2009). Discovering statistics using SPSS.

[68] Gibbs G (2007). Analyzing qualitative data.

[69] Kodner DL. Consumer-directed services: lessons and implications for integrated systems of care. Int J Integr Care. 2003; 10.5334/ijic.80. 10.5334/ijic.80 PMC1483950 16896379

[70] Johri M, Beland F, Bergman H. International experiments in integrated care for the elderly: a synthesis of evidence. Int J Geriat Psychiatry. 2003; 10.1002/gps.819. 10.1002/gps.819 12642892

[71] Giannini R, Petazzoni E, Savorani G, Galletti L, Piscaglia F, Licastro F, et al. Outcomes from a program of home care attendance in very frail elderly subjects. Arch Gerontol Geriatr. 2007; 10.1016/j.archger.2006.03.002. 10.1016/j.archger.2006.03.002 16750580

[72] Grundy E. Ageing and vulnerable older people: European perspectives. Ageing Soc. 2006; 10.1017/S0144686X05004484.

[73] Pijpers E, Ferreira I, Stehouwer CD, Nieuwenhuijzen Kruseman AC. The frailty dilemma. Review of the predictive accuracy of major frailty scores. Eur J Intern Med. 2012; 10.1016/j.ejim.2011.09.003. 10.1016/j.ejim.2011.09.003 22284239

[74] Knokke-Heist MD (2016). Agentschap Binnenlands Bestuur & Studiedienst Vlaamse Regering.

[75] Gent MD (2016). Agentschap Binnenlands Bestuur & Studiedienst Vlaamse Regering.

[76] Tienen MD (2016). Agentschap Binnenlands Bestuur & Studiedienst Vlaamse Regering.

